# Midline gallbladder makes a challenge for surgeons during laparoscopic cholecystectomy; case series of 6 patients

**DOI:** 10.1016/j.amsu.2019.02.005

**Published:** 2019-03-12

**Authors:** Ayad Ahmad Mohammed, Sardar Hassan Arif

**Affiliations:** University of Duhok, College of Medicine Azadi Teaching Hospital, 8 Nakhoshkhana Road, 1014, AM, Duhok City, DUHOK, Iraq

**Keywords:** Ectopic gall bladder, Midline gall bladder, Laparoscopic, Cholecystectomy, Gall stones

## Abstract

**Introduction:**

Gall bladder anomalies varies from variations in the size, site, duct systems, and shape. Abnormal location comprises the commonest one.

The presence of an ectopic gall bladder is estimated to occur in around 0.1–0.7% of individuals, it can be truly ectopic locating under the left lobe of the liver or just to the left of falciform ligament.

Gall stones are common disorder that may mandate cholecystectomy especially in symptomatic patients, surgery can be done laparoscopically safely in cases of abnormal anatomical position, but such cases may be at higher rate of complications especially if associated with other biliary tract anomalies.

**Methods:**

Six cases of symptomatic gall stones who underwent laparoscopic cholecystectomy included in this case series. During insertion of the telescope through the umbilical port, we found midline gallbladder under the falciform ligament instead being under right lobe of the liver. We did modification of the port sites by placing epigastric port in the left hypochonrdium.

**Results:**

In all the six cases the surgery had been done successfully laparoscopically without conversion to open technique. Follow up of the patients done for 2 months with no post-operative sequelae.

**Conclusion:**

Laparoscopic cholecystectomy for midline gall bladder is technically difficult. Modifying the port sites make the surgery easier. MRCP preoperatively, intraoperative cholangiography, or fluorescent cholangiography may be needed if there is any concern about biliary anomalies or for real time detection of biliary injuries.

## Highlights

• Anomalies of the gallbladder location may be encountered during surgery.
• It may be associated with anomalies of the round ligament or the portal venous system.
• Recognition of these anomalies has clinical importance when performing liver or gall bladder surgeries.

## 1 Introduction

Gall bladder anomalies are various ranging from variations in the size, site, anomalies of the duct systems, and shape [1].

Of these anomalies, variations of the location comprise the most common. The ectopic gall bladder may be located under the left lobe of the liver, being intrahepatic, in the falciform ligament, in the sub-capsular region over the anterior surface of the right lobe of the liver, in the anterior abdominal wall, in the suprahepatic region, or even retroplaced like in the retroperitoneum [2,3]. The condition of abnormal position of the gall bladder was first described by Hochstetter in 1886 in 3 anatomical specimens, and later in 1902 Kehr described this when he accidently found it during laparotomy [4]. Ectopic gall bladder is rare; estimated to occur in around 0.1–0.7% of the people, and may cause diagnostic confusion because patients may present with unusual site of the pain as in the epigastric or left hypochondrial regions [5].

It can be truly ectopic locating under the left lobe of the liver or just to the left of falciform ligament [6].

This anomaly may rarely be associated with congenital agenesis of the right lobe of the liver, duplication of the common bile duct or other anomalies of the portal venous system [[7], [8], [9]].

Sonographic diagnosis before surgery may be difficult, but CT scan and MRCP can help in better delineating the anatomy of the biliary system [1].

Gall stones are common disorder that may mandate surgical intervention especially in symptomatic patients or in patients having complications of gall stones.

The first successful cholecystectomy was done by the open technique which was in 1882 in Germany by Carl Langebuc. Later in 1985 and after 103 Erich Mühe performed the first successful laparoscopic cholecystectomy in Germany. After that until nowadays laparoscopic cholecystectomy has become the standard surgical procedure all over the world [10].

Cholecystectomy is usually done laparoscopically adopting the conventional 4 port technique or more recently surgery can be done using a single port, or two ports which are modifications of the conventional 4 port procedure [11,12].

The procedure of laparoscopic cholecystectomy has some complications such as bile duct injury, bowel injury, vascular injury, port site complications and adhesions [13].

Although the surgery can be done laparoscopically safely in cases of abnormal anatomical position of the gall bladder, but such cases may be at higher rate of complications especially if associated with other biliary tract anomalies, but no data are available about the rate of complications in such cases [14].

## 2 Methods

We present a series of 6 cases of midline gall bladder, these cases were encountered over a period of 5 years during elective operations for symptomatic gall stones.

The cases presented to the surgical consultation unit complaining from upper abdominal pain, in 4 cases the pain was felt in the right hypochondrial region and the remaining 2 cases felt the pain in the epigastrium that was radiated to the interscapular region.

Ultrasound of the abdomen showed gall stones with no signs on inflammation in all the cases with no any report about abnormal position of the gall bladder.

The operations done by 2 surgeons and no specific preoperative considerations were taken. All the six patients prepared for elective surgery, 2 were males and 4 females and they had no comorbid diseases.

Consent taken from all the patients postoperatively to be included and the ethical approval was exempted by the institution for reporting this case series.

Research registry done in accordance with the declaration of Helsinki at the Research Registry, UIN: 4603 at the 2nd of January 2019.

This work has been reported in line with the PROCESS criteria [15].

## 3 Results

**Fig. 1:**
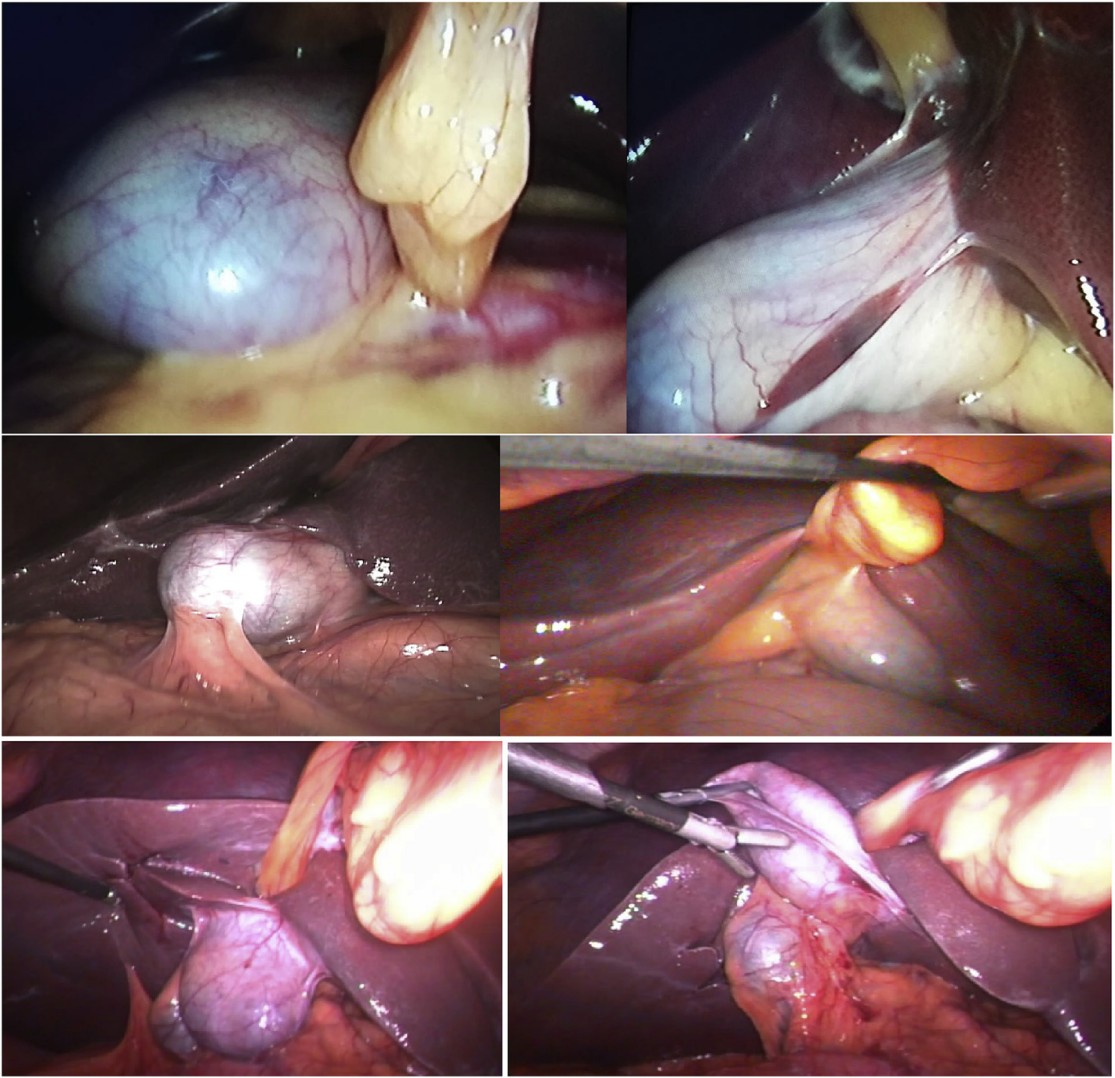
Intraoperative pictures of the 6 patients during laparoscopic cholecystectomy showing an abnormal position of the gall bladder being positioned in the midline under the falciform ligament.

During elective laparoscopic cholecystectomy and after insertion of the telescope through the umbilical port we discovered abnormal position of the gall bladder that was sited at the midline and left to the falciform ligament. Fig. 1.

**Fig. 2:**
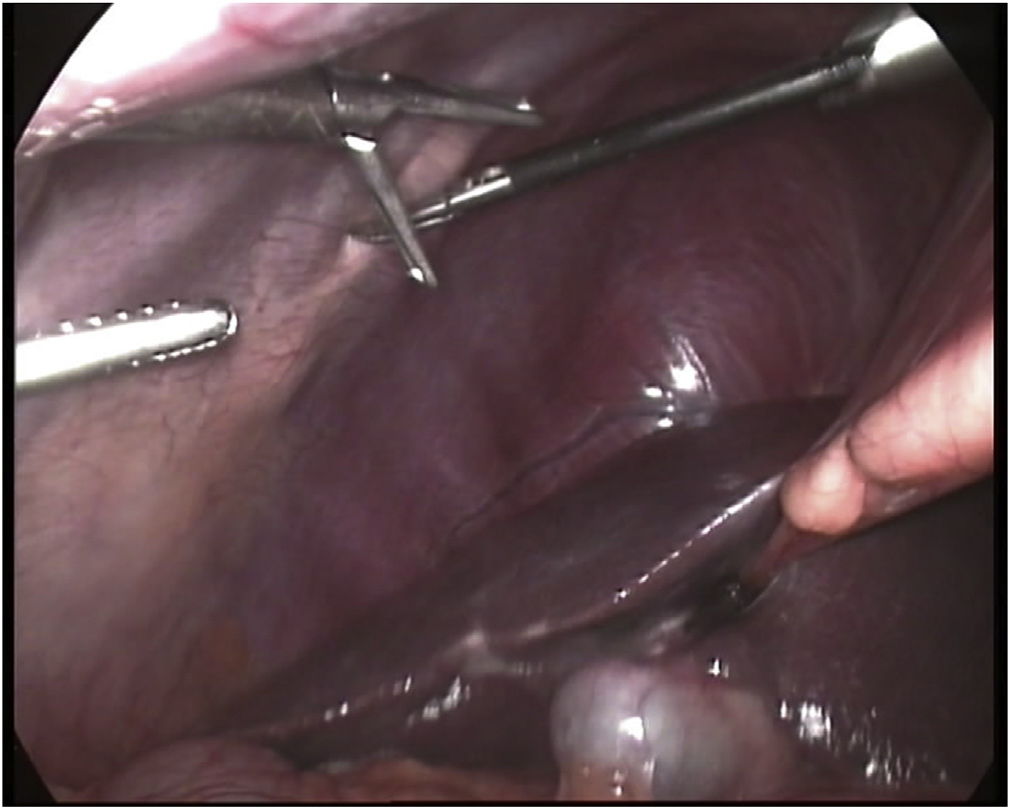
Intraoperative picture showing the position of the ports after placing them in the conventional positions which makes dissection very difficult.

In one case we introduced the ports in the conventional positions; i.e. one 10 mm port in the umbilicus for the telescope, one 11 mm port in the epigastric region in the midline, and other two 5 mm ports in the subcostal region in the midclavicular and anterior axillary lines respectively, we find a great difficulty in dissection especially by the epigastric port so we changed the position of that port to the left hypochonrdium. Fig. 2.

**Fig. 3:**
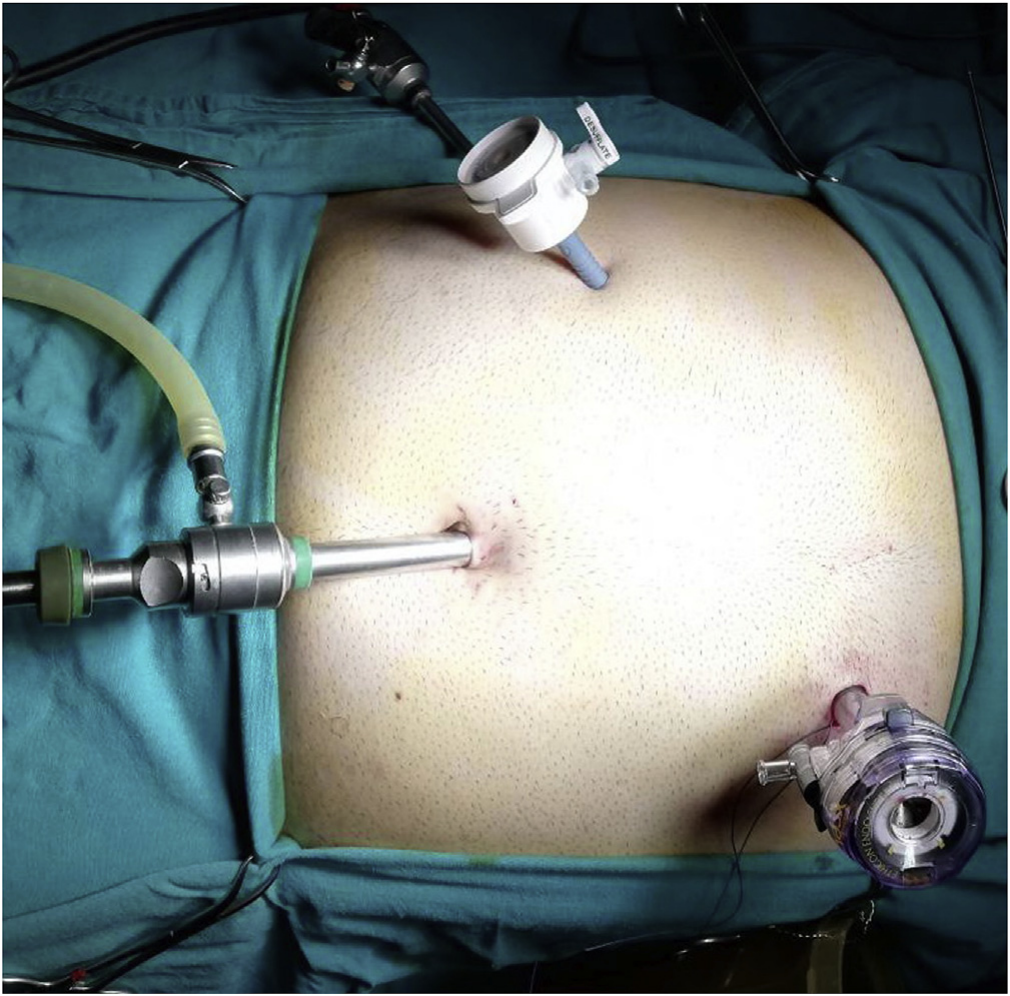
Showing the port sites during the operation placing one of the ports in the left hypochondrial region in the midclavicular line.

In the remaining 5 patients after insertion of the telescope and when we suspected abnormal position we next introduced the 5 mm port in the subcostal region in the midclavicular line, after elevation of the fundus of the gall bladder and identification of midline gall bladder we modified the position of the other port to the left hypochonrdium instead of placing it at the epigastric region which made the dissection and surgery easier. Fig. 3.

Dissection of the Calot's triangle done by removing the peritoneal coverings over the cystic duct and artery on the left side then on the right side using the 30° angled telescope. The critical view of safety; i.e. dissection of the gallbladder from its bed leaving the cystic duct and artery attached to it before the application of the clips and cutting, was considered the safest method during our work.

All the 6 cases had been operated successfully laparoscopically without intraoperative or postoperative complications during 2 months follow up. The only problem was increase operative time by 15–20 minutes compared to other patients in whom the gall bladder was normally sited.

There was no increase in the admission time and all patients discharged next day with no complications. No specific postoperative intervention taken.

## 4 Discussion

The clinical significance of this presentation is that because it may make confusion in clinical presentation and makes a real challenge during surgery. It will be technically very difficult or even may be impossible if the operation done with the port sites as in the conventional procedure. After insertion of the telescope through the umbilical port and if there is any suspicion of midline or ectopic position of the gall bladder we next placed the 5 mm port in the subcostal region in the midclavicular line and we used this port to introduce a grasper for elevation of the fundus of the gall bladder, when the anatomy become clear that it is a true midline gall bladder we did the modification of the conventional laparoscopic technique by placing other 10 mm port at the left hypochonrdium. This modification made the surgery easier and probably safer.

The presence of this variation can make identification of the Calot's triangle very difficult which needed frequent changing of the angle of view by the 30° telescope.

None of our cases was associated with situs inversus and regardless weather the is truly a left side gall bladder or due to abnormally located falciform ligament, this anomaly makes surgery technically more demanding and require modification of the port sites.

Most authors agree that preoperative identification of an abnormally placed gall bladder using the available imaging modalities is associated with less risk of complications especially biliary injuries. In most of the cases surgery can be done successfully laparoscopically. The exact rate of biliary complications in such cases is not accurately estimated, but no higher biliary complication rate has been reported [14,16,17].

Misidentification of the biliary anatomy is the most common cause of intraoperative biliary tract injuries, the use of intraoperative cholangiography had been used in the past for detection of biliary injuries, however, recently the use of fluorescent cholangiography using fluorescent agents excreted in the biliary system have been used for real time identification of such injuries [18].

The main limitation of our work is that all of the case were discovered during the operation, however if such cases are diagnosed preoperatively, more detailed imaging modalities like MRCP and intravenous contrast studies will help to detect other associated biliary and vascular anomalies.

## 5 Conclusions

Laparoscopic cholecystectomy for midline gall bladder is technically difficult, we do recommend the following points:

1. If there is any concern about the midline position after introduction of the telescope through the umbilicus, the next port to be placed should be the 5 mm port in the subcostal region in the midclavicular line and using this port for elevation of the fundus of the gall bladder to show the anatomy.
2. Changing the position of the epigastric port to the left hypochonrdium as a modification of the conventional technique.
3. Frequent movement of the 30° angled camera to visualize the Calot's triangle and showing the critical view of safety.
4. If the anatomy is not clear, we recommend doing intraoperative cholangiography or fluorescent cholangiography for intraoperative detection of biliary injuries.
5. If the diagnosis is done preoperatively we recommend adopting the French position (surgeon between the legs) with modification of the port sites for better ergonomy during the procedure.

## Ethical approval

No ethical committee approval was needed; consent have been taken from the patients to report their findings.

## Source of funding

There is no source of funding other than the authors.

## Author contribution

Study design: Dr Ayad Ahmad Mohammed and Dr Sardar Hassan Arif.

Data collections: Dr Sardar Hassan Arif.

Data analysis: Dr Ayad Ahmad Mohammed and Dr Sardar Hassan Arif.

Writing: Dr Ayad Ahmad Mohammed.

Final approval of the manuscript: Dr Ayad Ahmad Mohammed and Dr Sardar Hassan Arif.

## Conflicts of interest

No conflict of interest present.

## Research registration number

4603 Research registry, at 2/1/2019.

## Guarantor

Dr Ayad Ahmad Mohammed.

## Provenance and peer review

Not commissioned, externally peer reviewed.

## Appendix A Supplementary data

process_checklist process_checklist
