# Supplementary material for: Midline gallbladder makes a challenge for surgeons during laparoscopic cholecystectomy; case series of 6 patients
Source: Ann Med Surg (Lond). 2019 Mar 12;40:14–7. doi: 10.1016/j.amsu.2019.02.005 (PMC6423301; doi:10.1016/j.amsu.2019.02.005)
Supplement: process_checklist [file mmc1.docx]

| PROCESS Checklist | | | |
| --- | --- | --- | --- |
| Section | **Item** | **Checklist Description** | **Page Number** |
| Title | 1 | The words “case series” and the area of focus should appear in the title (e.g. disease, exposure/intervention or outcome). | 1 |
| Abstract | 2a | Introduction - what is the unifying theme of the case series. | 1 |
|  | 2b | Methods - describe what was done, how and when was it done and by whom. |  |
|  | 2c | Results - what was found. |  |
|  | 2d | Conclusion - what have we learned and what does it mean |  |
| Introduction | 3 | Explain the scientific background and rationale for the case series. What is the unifying theme - common disease, exposure, intervention and outcome, etc. Why is this study needed? | 1 & 2 |
| Methods | 4a | Registration and ethics - state the research registry number in accordance with the declaration of Helsinki - "Every research study involving human subjects must be registered in a publicly accessible database" (this can be obtained from; ResearchRegistry.com or ClinicalTrials.gov or ISRCTN). State whether ethical approval was needed and if so, what the relevant judgement reference was? | 3 |
|  | 4b | Study design - state the study is a case series and whether prospective or retrospective in design, whether single or multi-centre and whether cases are consecutive or non-consecutive. | 3 |
|  | 4c | Setting - describe the setting(s)and nature of the institution in which the patient was managed; academic, community or private practice setting? Location(s), and relevant dates, including periods of recruitment, exposure, follow-up, and data collection | 3 |
|  | 4d | Participants - describe the relevant characteristics of the participants (comorbidities, tumour staging, smoking status, etc). State any eligibility (inclusion/exclusion) criteria and the sources and methods of selection of participants. Describe length and methods of follow-up. | 3 |
|  | 4e | Pre-intervention considerations e.g. Patient optimisation: measures taken prior to surgery or other intervention e.g. treating hypothermia/hypovolaemia/hypotension in burns patients, ICU care for sepsis, dealing with anticoagulation/other medications and so on. | - |
|  | 4f | Types of intervention(s) deployed and reasoning behind treatment offered (pharmacological, surgical, physiotherapy, psychological, preventive) and concurrent treatments (antibiotics, analgesia, anti-emetics, nil by mouth, VTE prophylaxis, etc). Medical devices should have manufacturer and model specifically mentioned. | 3 |
|  | 4g | Peri-intervention considerations - administration of intervention (what, where, when and how was it done, including for surgery; anaesthesia, patient position, use of tourniquet and other relevant equipment, preparation used, sutures, devices, surgical stage (1 or 2 stage, etc). Pharmacological therapies should include formulation, dosage, strength, route and duration). | 3 |
|  | 4h | Who performed the procedures - operator experience (position on the learning curve for the technique if established, specialisation and prior relevant training). | 3 |
|  | 4i | Quality control - what measures were taken to reduce inter or intra-operator variation. What measures were taken to ensure quality and consistency in the delivery of the intervention e.g. independent observers, lymph node counts, etc | - |
|  | 4j | Post-intervention considerations e.g. post-operative instructions and place of care. Important follow-up measures - diagnostic and other test results. Future surveillance requirements - e.g. imaging surveillance of endovascular aneurysm repair (EVAR) or clinical exam/ultrasound of regional lymph nodes for skin cancer. | - |
| Results | 5a | Participants - reports numbers involved and their characteristics (comorbidities, tumour staging, smoking status, etc). | 3 |
|  | 5b | Any changes in the interventions during the course of the case series (how has it evolved, been tinkered with, what learning occurred, etc) together with rationale and a diagram if appropriate. Degree of novelty for a surgical technique/device should be mentioned and a comment on learning curves should be made for new techniques/devices. | - |
|  | 5c | Outcomes and follow-up - Clinician assessed and patient-reported outcomes (when appropriate) should be stated with inclusion of the time periods at which assessed. Relevant photographs/radiological images should be provided e.g. 12 month follow-up. | 4 |
|  | 5d | Where relevant - intervention adherence/compliance and tolerability (how was this assessed). Describe loss to follow-up (express as a percentage) and any explanations for it. | 4 |
|  | 5e | Complications and adverse or unanticipated events. Described in detail and ideally categorised in accordance with the Clavien-Dindo Classification. How they were prevented, diagnosed and managed. Blood loss, operative time, wound complications, re-exploration/revision surgery, 30-day post-op and long-term morbidity/mortality may need to be specified. | 4 |
| Discussion | 6a | Summarise key results | 4 |
|  | 6b | Discussion of the relevant literature, implications for clinical practice guidelines, how have the indications for a new technique/device been refined and how do outcomes compare with established therapies and the prevailing gold standard should one exist and any relevant hypothesis generation. | 4 |
|  | 6c | Strengths and limitations of the study | - |
|  | 6d | The rationale for any conclusions? | 4 |
| Conclusions | 7a | State the key conclusions from the study | 4 & 5 |
|  | 7b | State what needs to be done next, further research with what study design. | 4 & 5 |
| Additional Information | 8a | State any conflicts of interest | 5 |
|  | 8b | State any sources of funding | 5 |
